# Dual‐Mode Integrated Janus Films with Highly Efficient NaH_2_PO_2_‐Enhanced Infrared Radiative Cooling and Solar Heating for Year‐Round Thermal Management

**DOI:** 10.1002/advs.202206176

**Published:** 2023-01-13

**Authors:** Peng Yang, Jiajun He, Yanshan Ju, Qingyuan Zhang, Yipeng Wu, Zhengcai Xia, Liang Chen, Shaochun Tang

**Affiliations:** ^1^ School of Physics and Wuhan National High Magnetic Field Center Huazhong University of Science and Technology Wuhan 430074 P. R. China; ^2^ National Laboratory of Solid State Microstructures Collaborative Innovation Center of Advanced Microstructures Jiangsu Key Laboratory of Artificial Functional Materials College of Engineering and Applied Sciences Nanjing University Nanjing 210093 P. R. China; ^3^ Haian Institute of High‐Tech Research Nanjing University Jiangsu 226600 P. R. China

**Keywords:** dual‐mode film, Janus film, personal thermal management, radiative cooling, solar heating

## Abstract

The currently available materials cannot meet the requirements of human thermal comfort against the hot and cold seasonal temperature fluctuations. In this study, a dual‐mode Janus film with a bonded interface to gain dual‐mode functions of both highly efficient radiative cooling and solar heating for year‐round thermal management is designed and prepared. The cooling side is achieved by embedding NaH_2_PO_2_ particles with high infrared radiation (IR) emittance into a porous polymethyl methacrylate (PMMA) film during pore formation process, which is reported for the first time to the knowledge. A synergistic enhancement of NaH_2_PO_2_ and 3D porous structure leads to efficient radiant cooling with high solar reflectance (*R̅*_solar_ ≈ 92.6%) and high IR emittance (*ε̅*_IR_ ≈ 97.2%), especially the *ε̅*_IR_ value is much greater than that of the reported best porous polymer films. In outdoor environments under 750 mW cm^−2^ solar radiation, the dual‐mode Janus film shows subambient cooling temperature of ≈8.8 °C and heating temperature reaching ≈39.3 °C, indicating excellent thermal management capacity. A wide temperature range is obtained only by flipping the dual‐mode Janus film for thermal management. This work provides an advanced zero‐energy‐consumption human thermal management technique based on the high‐performance dual‐mode integrated Janus film material.

## Graphical abstract

A dual‐mode Janus film with bonded interface to gain radiative cooling and solar heating for year‐round thermal management. The cooling side with NaH_2_PO_2_ and 3D porous structure has a high infrared radiation (IR) emittance of 97.2%, which is much greater than that reported for the best porous polymer films. A wide temperature range of 48.1 °C is achieved by flipping Janus film.

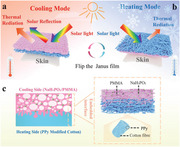

## 1 Introduction

In recent years, the worldwide climate is being accelerated warming and the climate extremes are increasing gradually.^[^ ^1^, ^2^, ^3^ ^]^ A wide temperature change in the outdoor environments not only influences body comfort but also has a direct impact on our health.^[^ ^4^, ^5^ ^]^ Usually various cooling/heating devices such as air conditioners, motor fans, and heaters are used in order to obtain a comfortable indoor environment. Obviously, these traditional cooling/heating devices require electricity, which inevitably requires large amounts of energy transformed from nonrenewable resources such as coal and thus results in the generation of large amounts of CO_2_ emissions.^[^ ^6^, ^7^, ^8^ ^]^ Recently, personal thermal management technology has been thought an effective technique that can effectively improve human thermal comfort with reduction of carbon emissions.^[^ ^9^, ^10^ ^]^ In particular, an advanced personal thermal management system based on expanding the ambient temperature range with high efficiency, and zero energy consumption has become one of the promising choices.^[^ ^11^, ^12^ ^]^

In an outdoor environment, the heat mainly comes from solar radiation (*λ* ≈ 0.25–2.5 µm) which is an important source of energy for local heating. The lack or excess of solar radiation usually leads to extreme outdoor environment.^[^ ^13^, ^14^ ^]^ Rational use of solar radiation is an ideal strategy to achieve zero energy consumption outdoor thermal management. In addition, radiating heat into cold outer space through atmospheric longwave infrared (LWIR) transmission windows (*λ* ≈ 8–13 µm) is another key factor.^[^ ^15^, ^16^, ^17^, ^18^ ^]^ It was demonstrated that the heat transfer could be controlled by adjusting the emissivity, transmissivity and reflectivity of the material to desired wavelength range.^[^ ^19^, ^20^ ^]^ In hot environments with strong solar radiation, effective passive radiative cooling (PRC) can be obtained without any additional energy input if the surface has high solar reflectance and high infrared emittance. In cold environments, self‐heating cannot be achieved by a low emissivity surface due to its inability to fully utilize the surrounding heat. High absorption of sunlight is required in cold environments with weak solar radiation. In recent years, dual‐mode Janus films with cooling and heating abilities have attracted extensive attention^[^ ^21^, ^22^, ^23^, ^24^, ^25^, ^26^ ^]^ due to their ability to achieve asymmetric properties in a single object with multiple functions. However, these reported Janus films have a weak physical bonding between the heating and cooling sides since they are usually pressed together, and especially their narrow temperature regulation range due to heating/cooling performance being sacrificed for dynamic tunability is still a key issue to limit practical applications.

A series of new cooling films were designed to achieve daytime radiative cooling. For example, multilayer photonic crystals, ^[^ ^27^, ^28^ ^]^ multilayer bulk materials,^[^ ^29^, ^30^ ^]^ metamaterials, randomly distributed particle structure, and porous structure show excellent PRC performance.^[^ ^31^, ^32^ ^]^ Especially, the polymer‐based porous structure with internally distributed particles is particularly appealing due to their low cost, simple manufacture, great scalability and superior cooling performance. A new structure consisting of 3D porous and single‐sided enriched microspheres, recently reported by us, shows excellent PRC capability of ≈6.2 °C for daytime sub‐ambient cooling. The performance equals or exceeds that at the time. It is obvious the shape, size, and spatial distribution all have an important influence on reflectance and emittance.^[^ ^33^ ^]^ Commonly used inorganic particles in polymers mainly include SiO_2_,^[^ ^29^ ^]^ TiO_2_,^[^ ^34^ ^]^ and Al_2_O_3_.^[^ ^35^ ^]^ However, other inorganic materials were seldom investigated, and the use of NaH_2_PO_2_ particles for improving radiative cooling has not been reported yet so far. NaH_2_PO_2_ has the strong *IR* emission peak at 8–13 µm to achieve ultra‐high emissivity, and thus is a potential radiative cooling material. Note that NaH_2_PO_2_ is stable in the dry state at relatively low temperatures, which assures long‐term stability.

As for solar heating, the key is developing photo‐thermal materials with high solar absorption and heat conversion efficiencies.^[^ ^36^, ^37^ ^]^ Black coatings including carbon nanotubes, ^[^ ^38^ ^]^ carbon black, graphene, ^[^ ^39^ ^]^ noble metal plasmonic nanoparticles^[^ ^40^ ^]^ and multilayer selective solar absorbing material^[^ ^41^ ^]^ were already applied for outdoor solar heating. However, high cost and complex fabrication process limited their practical applications. Polypyrrole (PPy) was extensively used in photothermal therapy and solar vapor generation due to its efficient light absorption and high stability.^[^ ^42^ ^]^ Additionally, PPy‐modified cotton (PMC) can be controllably prepared at a large scale and thus is a competitive as a photothermal material for human thermal management.

In this work, a dual‐mode Janus film with a bonded interface and dual‐mode functions of both highly efficient radiative cooling and solar heating for year‐round thermal management were designed and prepared. The cooling side with highly enhanced radiant cooling ability was achieved by embedding NaH_2_PO_2_ particles with high infrared radiation (IR) emittance into a porous PMMA film. We found that the infrared emission peaks of NaH_2_PO_2_ and PMMA at the atmospheric window were complementary. The dispersed NaH_2_PO_2_ particles lead to broadband radiative cooling, together with enhanced diffuse reflection endow the PMMA/NaH_2_PO_2_ film with high reflectance. By changing the side of Janus film towards the environment, radiative cooling or solar heating function is achieved to meet body thermal comfort at different ambient temperatures. The Janus film prevents drastic temperature changes on the skin and creates a comfortable temperature for year‐round thermal management.

## 2 Results and Discussion

**Figure 1:**
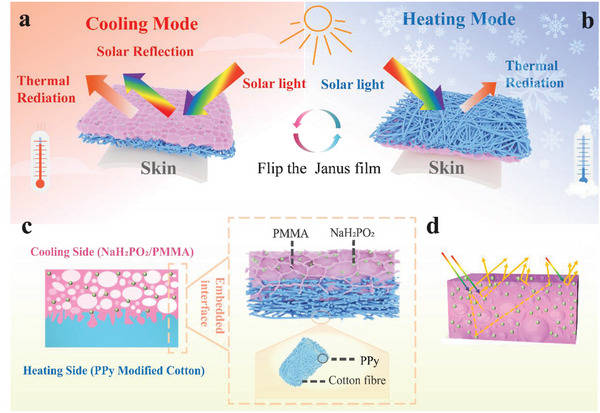
Schematic diagram of dual‐mode Janus film with cooling and heating side. Working principle and the structure features of the Janus film in a) cooling mode and b) heating mode. c) The cross‐sectional diagram of the dual‐mode Janus film. d) Schematic diagram of sunlight scattering principle of a three‐dimensionally porous structure containing microsized particles.

**Figure** **1** shows the schematic diagram of Janus film covering the human skin surface for temperature regulation. The cooling or heating mode is realized by selecting a side of the Janus film to face the environment. In hot environments (Figure 1a), the cooling side of Janus film with PMMA/NaH_2_PO_2_ porous structure is oriented toward the sky. It has a high solar reflectance and thus absorbs the least amount of energy from the sunlight. At the same time, the high infrared radiation makes it radiate heat to cold outer space through the atmospheric transparency window (8‐13 µm). By increasing the reflectance in the sunlight band and the emittance in the infrared band, Janus films achieve daytime radiative cooling. When being in a cold environment (Figure 1b), the black heating side with a high solar absorbance is directed toward to sky. The heating side absorbs a large amount of sunlight and converts it to the heat energy.

The cross‐sectional diagram of Janus film is shown in Figure 1c, which depicts the cooling side and heating side and their combination in detail. The cooling and heating sides are combined by the PMC fibers embedded in the polymer network to form an integrated Janus film. NaH_2_PO_2_ doped porous PMMA film (PMMA/NaH_2_PO_2_) as the cooling side. The PMMA and NaH_2_PO_2_ are excellent passive radiative cooling materials due to their inherent characteristics of high emittance of 8–13 µm. In addition, the film has high solar reflectance because NaH_2_PO_2_ particles and the porous structure can effectively reflect (scatter) sunlight. The porous structure and NaH_2_PO_2_ particles contribute to reinforcing the total scattering efficiency due to the different refractive indices, resulting in a highly reflective interface. And the probability of infrared absorption/emission is increased by multiple diffuse reflections in the disordered porous structure with different incidence angles (Figure 1d). The heating side is composed of tightly stacked cotton fibers, whose surface is in‐situ grown by PPy with favorable photo‐thermal conversion performance.^[^ ^43^ ^]^ The contact surfaces on the cooling and heating side are irregularly embedded. The layer of PMC fibers on the contact surface is surrounded by PMMA, forming a strongly embedded interface.

**Figure 2:**
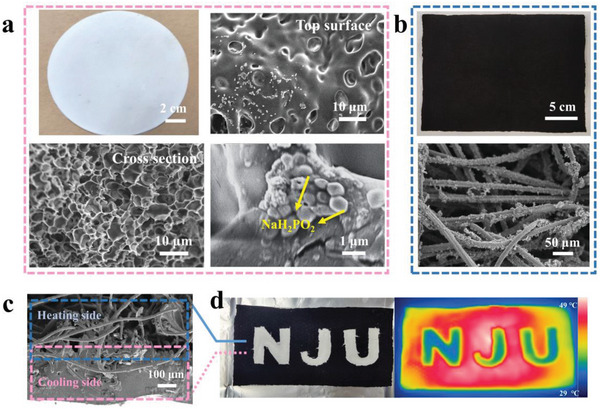
Morphology of the dual‐mode Janus films. a) Digital Photo and SEM images of cooling side, including a top surface and cross‐section. b) Digital Photo and SEM images of heating side. c) Cross section at the combination of cooling side and heating side. d) Digital and IR photos of a tailored dual‐mode Janus film with white “NJU” patterns on the black side.

**Figure** **2**a shows the digital photo and SEM of the cooling side, which presents a white appearance. The top surface and cross‐section show a densely distributed irregular porous structure on the cooling side, and spherical NaH_2_PO_2_ particles with a diameter of ≈1 µm are deposited on the porous wall. In addition, the EDS elemental mapping also proves the existence of NaH_2_PO_2_ (Figure S1, Supporting Information). The pores are formed due to solvent precipitation, and the pore size distribution is analyzed with Nano Measurer 1.2. The results show that the porous size is unimodally distributed, with a broad distribution centered at ≈4 µm. Widely distributed porous can effectively reflect (scatter) sunlight and help to obtain high solar reflectivity.^[^ ^30^ ^]^ The morphology of the heating side of Janus film is shown in Figure 2b. The black appearance shows strong sunlight absorption capacity, indicating a strong light absorption capacity. The microstructure of SEM shows that the heating side is composed of fibers with a diameter of ≈20 µm staggered, and PPy particles are uniformly loaded on the fiber surface.

The cross‐section SEM image of Janus film is shown in Figure 2c. The fibers on the upper part as the heating side and the polymer membrane (average thickness 200 µm) on the lower part as the cooling side can be clearly seen. The middle part of the Janus film shows some of the PMC fibers embedded in the PMMA. There is a strong color difference between the white cooling side and the black heating side, so a dual‐mode Janus film with “NJU” pattern is customized (Figure 2d). A very clear “NJU” pattern was observed within 3 seconds of placing it under a solar simulator, as the white area cools down and the black area of the film heats up rapidly, resulting in a large temperature difference between the two areas.

**Figure 3:**
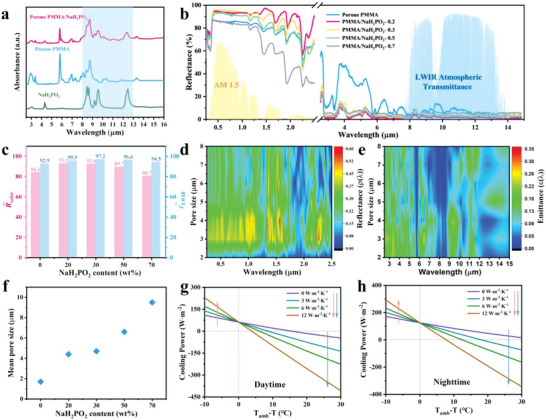
Optical properties of the cooling side. a) Absorbance spectra of NaH_2_PO_2_, PMMA, and PMMA/NaH_2_PO_2_ films were measured with ATR‐FTIR spectroscopy. b) Spectral reflectance, the normalized ASTM G173 Global solar spectrum and the LWIR atmospheric transparency window. c) The average solar reflectance *R̅*_solar_ and average infrared emittance ${\bar{\varepsilon }}_{\mathrm{IR}}$. d,e) Simulated reflectance and emissivity of the PMMA films with different pore sizes. f) Variation of statistical average aperture with NaH_2_PO_2_ content. The calculated net cooling power of PMMA/ NaH_2_PO_2_‐0.3 during the g) daytime and h) nighttime.

High solar reflectivity and high infrared emittance are necessary to achieve daytime radiative cooling. As shown in **Figure** **3**a, the absorbance spectra of pristine PMMA and NaH_2_PO_2_ measured with attenuated total reflectance‐Fourier transform infrared spectroscopy (ATR‐FTIR) show strong infrared absorption within 8–13 µm. Moreover, the addition of NaH_2_PO_2_ fills the emittance valleys of PMMA in the 11–13 µm region, resulting in enhanced emission of the composite film in the atmospheric window. By adjusting the weight ratio of NaH_2_PO_2_ microspheres in the cooling layer, the tunability of the optical properties of the cooling layer was systematically investigated. Figure 3b shows the optical reflectance of the PMMA/NaH_2_PO_2_ films in the solar and mid‐infrared regions. The average solar reflectance (*R̅*_solar_) of pure porous PMMA films relies on the scattering of sunlight by the pores to obtain (≈84%), and the addition of NaH_2_PO_2_ particles significantly improves the reflectivity of the film. With an increase of NaH_2_PO_2_ content, *R̅*_solar_ has an obvious trend of increasing and then decreasing and the maximum value of 93.1% was achieved at PMMA/NaH_2_PO_2_‐0.2, as shown in Figure 3c. All films have a high average infrared emittance (*ε̅*_IR_) of PMMA, increasing the *ε̅*_IR_ in the 8–13 µm range from 93% for PMMA to more than 96% for PMMA/NaH_2_PO_2_ in the LWIR transparency window. The high *ε̅*_IR_ is attributed to the synergistic effect of the C—O—C bonds in PMMA and the P=O, P—H bonds in NaH_2_PO_2_, which improves the infrared radiation performance of PMMA/NaH_2_PO_2_ film and is more conducive to radiating energy outside which makes the film have strong thermal emission properties.

To investigate the size effect of the pores on the optical properties, finite‐difference time‐domain (FDTD) was used to simulate the spectrally selective property.^[^ ^44^ ^]^ The solar reflectance of PMMA films reaches its maximum at a micropore diameter of 3–4 µm (Figure 3d). Similarly, the thermal emissivity in the 8–13 µm range is consistent with the solar reflectance associated with the pore size in PMMA films (Figure 3e). Accordingly, a pore size close to 4 µm is important for PMMA films to achieve efficient passive radiative cooling performance. The average pore size increases from 2 to 10 µm when the NaH_2_PO_2_ content increases from 0 to 70 wt% (Figure 3f). The pore size close to 4 µm of PMMA/NaH_2_PO_2_‐0.2 and PMMA/NaH_2_PO_2_‐0.3 can effectively scatter all wavelengths of sunlight, as confirmed by FDTD, thus demonstrating high solar reflectivity. The spectral characteristics of high solar reflection and high infrared emittance contribute to the heat exchange between the polymer and the atmosphere to achieve cooling. To sum up, PMMA/NaH_2_PO_2_‐0.3 is selected as the optimal cooling side to achieve the strongest radiative cooling performance. It has a high *R̅*_solar_ of 92.6% and an ultrahigh *ε̅*_IR_ of 97.2%, especially the *ε̅*_IR_ value is much greater than that of the reported best porous polymer films (Table S1, Supporting Information). Although solar reflectance of the cooling side is not outstanding when compared with those with multiscale pores, the ultrahigh infrared emissivity plays a decisive role for the large enhancement of radiative cooling.

The cooling performance such as net cooling power and temperature decrease is numerically analyzed, which is based on the measured data of reflection spectrum and transmission spectrum and calculated by the radiative cooling theoretical model.^[^ ^45^ ^]^ More details of this model are given in the “Experimental” section. The *P*_net_ is defined as the net cooling power at ambient air temperature (*T*_amb_ ‐ *T* = 0), which is only affected by radiant heat. And the Δ*T*_max_ is defined as the maximum achievable cooling temperature (*ΔT*_max_ = (*T*_amb_ ‐ *T*_s_)_max_) at *P*_net_ = 0. As can be seen from Figure 3g,h, the Δ*T*_max_ of PMMA/NaH_2_PO_2_‐0.3 decreases with the increasing *h_c_* value due to the nonradiative heat contributing to the power input when *T*_amb_ exceeds *T*_s_. The cooling performance of materials is largely affected by nonradiative heat transfer (such as atmospheric humidity, wind speed, geographical location, etc.).^[^ ^46^ ^]^ Moreover, with a heat transfer coefficient *h_c_* = 3 W m^−2^ K^−1^, the Δ*T*_max_ can still reach ≈8.6 °C and 17.5 °C during the daytime and nighttime, respectively. The differences in *P*_net_ and Δ*T*_max_ between daytime and nighttime display the importance of the high solar reflectance to daytime radiative cooling. Furthermore, the calculated *P*_net_ and Δ*T*_max_ of a series PMMA/NaH_2_PO_2_ films during daytime and nighttime are shown in Figures S4 and S5 (Supporting Information). A similar cooling effect can be achieved at nighttime due to the high and similar IR emittance. The *P*_net_ and Δ*T*_max_ are strongly correlated with the reflectance of the material during the daytime, which has a trend consistent with that of Figure 3b. The PMMA and PMMA/NaH_2_PO_2_‐0.7 cannot obtain the cooling effect in the daytime due to the low solar reflectivity. Most notably, these results verify that the NaH_2_PO_2_ embedded PMMA porous structure, especially PMMA/NaH_2_PO_2_‐0.3, has enhanced solar reflectance and high infrared emittance, which plays an important role in realizing radiative cooling.

**Figure 4:**
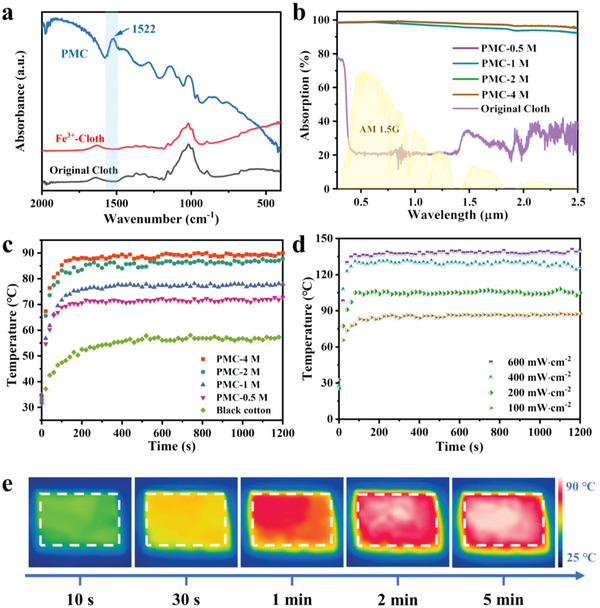
Solar heating properties of the heating side. a) ATR‐FTIR spectra of the original cloth, ferric treated cotton, and PMC. b) Solar spectral irradiance (AM 1.5 G) and absorption curves of PMC with different Fe^3+^ concentrations. c) The temperature versus time curves of PMC with different Fe^3+^ concentrations under 1 sun. d) The temperature versus time curves of PMC‐2 M under different light intensities. e) Infrared images of PMC‐2 M under 1 sun.

Solar heating properties of the heating side are shown in **Figure** **4**. The chemical composition of PMC was determined by ATR‐FTIR spectroscopy. Compared to original cotton and iron‐modified cotton, there is a characteristic peak at 1542 cm^−1^, which strongly proves the successful modification of PPy on the cotton surface.^[^ ^43^, ^47^, ^48^ ^]^ The concentration of Fe^3+^ (FeCl_3_/H_2_O), which forms ligand interactions with cotton fabrics, was varied to produce a series of PMC, to optimize the photothermal properties of PMC. As displayed in Figure S4 (Supporting Information), with the increase of Fe^3+^ concentration ranging from 0.5 to 4 m, increasing PPy was formed on the cotton surface. In particular, the surface morphology of PMC‐4 M became very rough, due to the large accumulation of PPy particles. Absorbance is a key factor affecting the solar‐thermal energy conversion efficiency of material, and the ultraviolet–vis (UV–vis) near‐infrared (NIR) spectra of PMC are shown in Figure 4b. Compared with the low absorbance of original cotton, PMC showed extremely high absorbance over the whole spectral range, indicating its excellent broad‐spectrum absorption performance. The absorbance of PMC increased from 96% to 98% with the increase of Fe^3+^ concentration. However, the absorbance remained stable when the Fe^3+^ concentration was greater than 3 m.

The photothermal conversion performance of PMC is quantified by the temperature rise from room temperature to surface equilibrium temperature. The illuminance was simulated by a solar simulator, and the surface temperature was measured by an infrared camera. The attainable temperature of PMC with different Fe^3+^ concentrations under 1 sun was shown in Figure 4c, which represented a gradual temperature increase and finally tended to be a stable value. The black cotton rises from room temperature to 55 °C and remains stable within 400 s. In contrast, PMC rises to more than 70 °C and reaches equilibrium within 120 s. The high temperature rise of over 40 °C and the short rise time demonstrate excellent photothermal conversion of PMC. Compared with other black materials, our PMC exhibits much superior solar heating performance due to its ultra‐high light absorption (98%) and fast photothermal conversion (Table S2, Supporting Information).

PMC‐2 M with uniform morphology and excellent performance was preferably selected in further experiments. To simulate environments with large temperature changes, such as summer and winter, PMC‐2 M was placed under *I*_solar_ of 100–600 mW cm^−2^ and record its temperature–time curve as shown in Figure 4d. It can be seen that the maximum temperature and the heating rate are highly dependent on the light intensity. The surface temperature of PMC‐2 M can rise rapidly from room temperature to over 50 °C under 100 mW cm^−2^ in a short time, and the temperature remains stable after 2 min (Figure 4e).

To demonstrate the cooling and heating ability of the dual‐mode Janus film, the temperature contrast between white cotton, black cotton and two side of Janus film was measured. Placed the sample under the 100 mW cm^−2^ solar simulator, and the surface temperature was recorded with an infrared camera. As shown in Figure S7 (Supporting Information), the surface temperatures of the four samples are similar at 0 s. After exposure to a solar simulator with an intensity of 1 sun, the IR image changes significantly. After about 120 s of illumination, it is seen that the heating and cooling sides exhibit the highest and lowest temperatures, respectively, showing excellent temperature regulation relative to conventional cotton.

**Figure 5:**
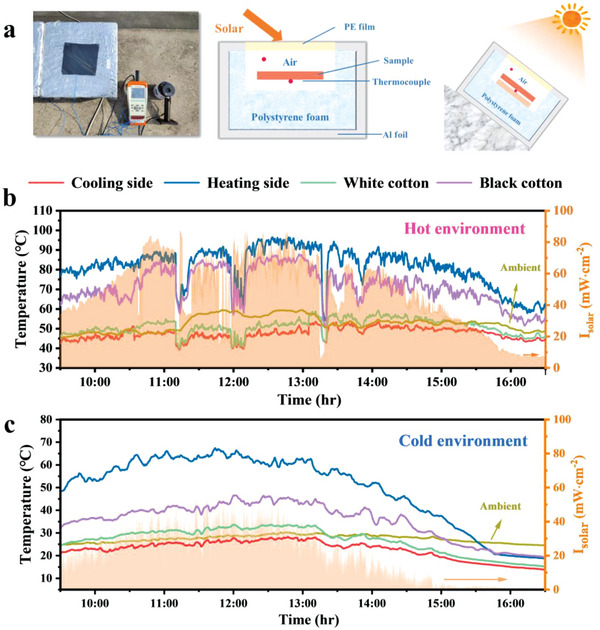
Outdoor thermal measurement (cooling and heating side) of the Janus film. a) Photos and schematic of the setup. The real‐time temperatures of the samples (line) and solar irradiance power density in b) hot environment (August 7, 2022) and c) cold environment (November 19, 2022) in Nanjing, China.

Radiative cooling performance in real‐world applications is substantially affected by the geographical regions and climates. We exposed experimental setups to the sky on a building roof in Nanjing city, China (32°2′16″N, 118°52′26″E) (**Figure** **5**a). Our measurement apparatus uses polystyrene foam to isolate the ambient heat, Al foil to reflect the sunlight, and a PE film on top to reduce heat convection.^[^ ^49^ ^]^ The results of the 7 h test at noon in summer with hot environment are shown in Figure 5b. During continuous testing, the ambient temperature is basically kept above 50 °C, The ambient temperature remained above 50 °C during the continuous test, accompanied by strong sunlight. Compared with the ambient temperature, the cooling side achieves a temperature reduction of ≈8.8 °C under *I*_solar_ of 75 mW cm^−2^ at 12:15–13:15, which is due to the high solar reflectance and high infrared emittance. In contrast, the heating side achieves a super ambient temperature rise of ≈49.5 °C under solar irradiation due to its high absorptivity and excellent photothermal conversion. The Janus film realizes that the cooling side is 4.3 °C lower than white cotton and the heating side is 9.6 °C higher than black cotton on average. A depression of the solar intensity curve corresponds to a sky environment with cloud cover. The temperature of the film changes rapidly as the solar intensity fluctuates, especially on the heated side with ultra‐high solar absorption, reflecting the excellent photothermal response efficiency. In cold environment, it can still achieve a solar ambient cooling temperature of ≈4.2 °C and a super ambient heating temperature of ≈45.5 °C under *I*_solar_ of 29 mW cm^−2^. This further demonstrates the excellent thermal management performance of the dual‐mode Janus films.

**Figure 6:**
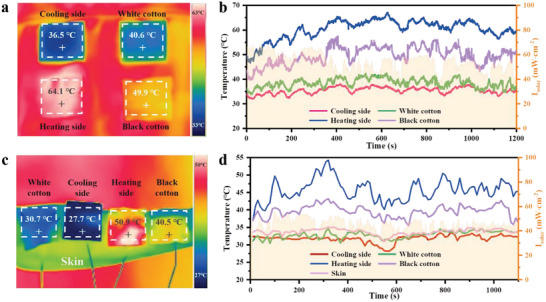
Outdoor personal thermal management performance of the dual‐mode Janus films (April 11, 2022, Nanjing, China). a) IR image of cotton and dual‐mode Janus films over human clothing and b) corresponding surface temperature, c) IR image of cotton and dual‐mode Janus films over bare leaking human skin, and d) corresponding temperature at the bottom of the film.

To be used for outdoor human thermal management, the dual‐mode Janus film is crucial for the thermoregulatory properties of human skin. Therefore, the Janus film was used to cover traditional human clothes (**Figure** **6**a) and bare human arms (Figure 6c) and to record the temperature respectively. The real‐time surface temperature record of Janus film covered on clothing is shown in Figure 6b. Under the direct sunlight of *I*_solar_ ≈60 mW cm^−2^ at 11:00–12:00 am, the cooling side has been maintaining the lowest temperature in all samples due to its good radiative cooling performance. Meanwhile, the heating side increases temperature rapidly when exposed to sunlight, thus showing a good solar heating effect. Further, the Janus film was placed directly over the arm skin to prove its thermal management performance for the skin. The temperature was monitored in real time by a thermometer placed at the bottom of the film. As shown in Figure 6d, the bottom temperature on the cooling side was ≈2 °C lower than the bare‐leakage skin temperature, while the white cotton fabric temperature was at the same level as the skin temperature. Correspondingly, the bottom temperature of the heating side is ≈15 °C and ≈5 °C higher than the temperature of the bare skin and the black cotton. The Janus films achieved excellent thermal management performance of cooling in hot environment and heating in cold environment by flipping the films. Switching between heating and cooling can even take place at different times. These results show that it has great potential for commercial application in various complex scenarios.

Thermal conductivities of the cooling and heating layers were measured using the transient flat plate heat source method, and they are 0.089 and 0.112 W m^‐1^ K^‐1^, respectively (Table S3, Supporting Information). Heat conduction is more important than heat radiation between the human skin and the inner surface of textiles. The asymmetric thermal conductivity contributes to achieve a wide temperature range for thermal management. The flexibility of Janus film was assessed by bending and folding. As shown in Figure S8a (Supporting Information), the film retains its original shape and does not break after bending and folding diagonally to the inside or outside. The strong adhesion between cooling and heating sides can load or lift a heavy object about 1 kg, as shown in Figure S8b (Supporting Information). These advantages the dual‐mode Janus film with a bonded interface promise good prospects for wearable scenarios.

As shown in Figure S9a (Supporting Information), the surface contact angle of the cooling layer was 131° which proves its high hydrophobicity. Even after immersing the cooling layer in an aqueous solution for 24 and 48 h, some strong characteristic absorption peaks corresponding to NaH_2_PO_2_ are still present in IR absorption spectra, as Figure S9b (Supporting Information). This is because NaH_2_PO_2_ particles are embedded in the porous walls of PMMA film, the hydrophobic surface prevented the intrusion of aqueous solutions and thus delayed its deliquescence. Further, since the high emissivity of NaH_2_PO_2_ in the atmospheric window is provided mainly by the vibration of H_2_PO_2_^−^, the generation of insoluble core–shell structures or phosphates could be a better solution for long‐term and wider applications. Adding inorganic particles with high infrared emissivity as an expandable method is expected to be applied for enhanced radiative cooling in a wide range of material structures and scenarios.

## 3 Conclusions

In summary, we designed and manufactured a dual‐mode Janus film that concentrates the opposite requirements of heating and cooling. The Janus film with embedded interface between two sides and achieve efficient thermal management in both hot and cold environments by flipping. The porous structure of NaH_2_PO_2_ doped PMMA as cooling side can efficiently reflect sunlight and emit infrared radiation, showing ultra‐high *R̅*_solar_ and *ε̅*_IR_ values of ≈92.6% and ≈97.2%, respectively. Especially the IR emittance is greater than that for the best reported porous polymer with randomly distributed porous. The heating side of PMC has a solar absorptance of 98% and allows for efficient photothermal conversion. For practical applications, the Janus film provides excellent thermoregulation capacity in outdoor environments, it can actually achieve a solar ambient cooling temperature of ≈8.8 °C and a super ambient heating temperature of ≈39.3 °C under *I*_solar_ of 75 mW cm^−2^. The strong cooling performance is mainly attributed to the incorporation of unreported NaH_2_PO_2_ particles, which in effective synergy with the porous structure can effectively increase the solar reflectance of the system and improve the radiation output. The Janus films with integrated heating and cooling have great potential in applications and offer new possibilities for personal thermal management due to their low cost and easy to manufacture approach.

## 4 Experimental Section

### Fabrication of Janus Films: Fabrication of PPy Modified Cotton (Heating Side)

The cotton fabric was rinsed thoroughly with acetone and ethanol to remove oily residue, then washed with deionized water and dried. First, the obtained clean cotton fabric was immersed in different concentrations of FeCl_3_·6H_2_O solution (0.5–4 mol L^‐1^) for 30 min to absorb enough Fe^3+^. In the second step, the cotton fabric attached with Fe^3+^ was immersed in pyrrole solution (0.2 mol L^‐1^) for 30 min. After oxidative polymerization, the cotton fabric was washed with deionized water many times to remove any Fe^3+^/Fe^2+^ and unstable polymer. Finally, the PMC was obtained by drying it in an oven for 2 h. The obtained PMC at different concentrations of FeCl_3_·6H_2_O were denominated as PMC‐0.5 M, PMC‐1 M, PMC‐2 M, and PMC‐4 M.

### Fabrication of NaH_2_PO_2_ Doped Porous PMMA Film (Cooling Side)

Add a certain amount of NaH_2_PO_2_ particles into 6 g DMF and stir magnetically at 35 °C for 1 h to form a uniform dispersion. Immediately, 1 g PMMA particles were added to the mixed solution and stirred at 35 °C for 4 h. After it was completely mixed and dissolved, the dispersed liquid was injected onto the glass substrate. After the solvent was completely precipitated and evaporated, the film was stripped from the surface of the glass base to obtain an independent film. The cooling layers with different NaH_2_PO_2_ content (based on the NaH_2_PO_2_/PMMA mass ratio: 0.2, 0.3, 0.5, and 0.7) were prepared, which are referred to as PMMA/NaH_2_PO_2_‐0.2, PMMA/NaH_2_PO_2_‐0.3, PMMA/NaH_2_PO_2_‐0.5, and PMMA/NaH_2_PO_2_‐0.7, respectively.

### Fabrication of Janus Films

The NaH_2_PO_2_ doped PMMA dispersion was evenly distributed on the glass substrate, and then the prepared PMC was covered on its upper surface. After waiting for the solvent to evaporate completely, peel the composite film from the glass plate.

### Material Characterizations

The morphology was characterized by scanning electron microscopy (SEM) images obtained on Hitachi S4800 (Japan) with an energy‐dispersive X‐ray spectrometer (EDX). To measure the photothermal conversion performance indoors, a xenon lamp light source (CEL‐PE300L‐3A) is used to simulate sunlight and irradiate the sample, and the surface temperature is recorded by an infrared thermal imager (FOTRIC 320). The thermal conductivities of samples were measured using the transient flat plate heat source method by Hot Disk TPS 2500S.

The reflection spectrum of 0.25–2.5 µm was measured by a UV–Vis–NIR spectrophotometer (Shimadzu UV‐3600) attached to a diffuse integrating sphere (ISR‐3100). The emittance of the mid‐infrared band (2.5–15 µm) is determined by a Fourier infrared (FTIR) spectroscopy (Nicolet iS50R) equipped with a gold integrating sphere (PIKE Technologies) by reflection method, and the test window is zinc selenide window. According to Kirchhoff's law, due to the opacity of the object, emittance = 1‐reflectance. The vibration absorption of groups in the material structure is also characterized by FTIR. Outdoor cooling performance was measured using a self‐assembled radiant refrigeration performance test device in Nanjing, China (32°2′ 16″ N, 118°52′ 26″ E).

The average reflectance ${\bar{R}}_{\mathrm{solar}}\left(\theta \right)$ and average infrared emittance ${\bar{\varepsilon }}_{\mathrm{LWIR}}$ of materials are defined by Equations (1) and (2) respectively:

(1) $$\begin{array}{c} {\bar{R}}_{\mathrm{solar}}\;\left(\theta \right)=\frac{\int_{0}^{\infty }I_{\mathrm{solar}}\left(\lambda \right)R_{\mathrm{solar}}\left(\theta ,\lambda \right)d\lambda }{\int_{0}^{\infty }I_{\mathrm{solar}}\left(\lambda \right)d\lambda }\; \end{array}$$

(2) $$\begin{array}{c} \;{\bar{\varepsilon }}_{\mathrm{LWIR}}=\frac{\int_{8\mu m}^{13\mu m}I_{\mathrm{BB}}\left(T,\lambda \right)\varepsilon \left(T,\lambda \right)d\lambda }{\int_{8\mu m}^{13\mu m}I_{\mathrm{BB}}\left(T,\lambda \right)d\lambda }\; \end{array}$$

### Finite‐Difference‐Time‐Domain Simulations

This study is based on the FDTD method for numerical research. The boundary conditions are set as follows: periodic in the horizontal direction (periodic boundary condition, PBC) and absorption in the vertical direction (perfectly matched layer, PML). A plane wave source illuminates the structure. The wavelength of solar research is defined as 0.25–2.5 µm, and the wavelength of radiative cooling research is defined as 2.5–15 µm. To investigate the effect of pore size on reflectivity performance, different sizes of random porous with diameters ranging from 2 to 8 µm were simulated, respectively. The pore distribution density was the same (68%) for each model. The appropriate mesh size was determined to achieve a good trade‐off between computer memory requirements and simulation time. Convergence tests were carefully performed.

### Calculations of the Cooling Power of the Passive Radiative Cooler

In an open environment, the material will emit heat through the surface, and the absorbed heat includes the heat from solar radiation (*P*_solar_), ambient radiation (*P*_amb_) and heat transfer by conduction and convection due to temperature differences (*P*_conv + cond_). The net cooling power refers to the difference between the radiated power and the absorbed power, expressed as,

(3) $$\begin{array}{c} \;P_{\mathrm{cool}}=P_{\mathrm{rad}}\;-P_{\mathrm{amb}}-P_{\mathrm{solar}}-P_{\mathrm{conv}+\mathrm{cond}} \end{array}$$

At night, due to the disappearance of solar radiation, the expression of net cooling power can be simplified as

(4) $$\begin{array}{c} \;P_{\mathrm{cool}}=P_{\mathrm{rad}}\;-P_{\mathrm{amb}}-P_{\mathrm{conv}+\mathrm{cond}} \end{array}$$

Specifically, the radiated energy through the cooling radiator:

(5) $$\begin{array}{c} \;P_{\mathrm{rad}}=\;A\int d\Omega \cos\theta \int_{0}^{\infty }d\lambda I_{\mathrm{BB}}\left(T_{s},\lambda \right)\varepsilon \left(\lambda ,\theta \right) \end{array}$$

the absorbed energy from solar radiation:

(6) $$\begin{array}{c} \;P_{\mathrm{amb}}=\;A\int d\Omega \cos\theta \int_{0}^{\infty }d\lambda I_{\mathrm{BB}}\left(T_{\mathrm{amb}},\lambda \right)\varepsilon \left(\lambda ,\theta \right)\varepsilon_{\mathrm{atm}}\left(\lambda ,\theta \right) \end{array}$$

the absorbed energy from solar radiation:

(7) $$\begin{array}{c} \;P_{\mathrm{solar}}=\int_{0}^{\infty }d\lambda \;\in \left(\lambda ,\theta_{\mathrm{sol}}\right)I_{\mathrm{AM}1.5}\left(\lambda \right) \end{array}$$

The lost energy due to convection and conduction

(8) $$\begin{array}{c} P_{\mathrm{conv}+\mathrm{cond}}\;\left(T_{s},T_{\mathrm{amb}}\right)=\;Ah_{c}\left(T_{\mathrm{amb}}-T_{s}\right) \end{array}$$

where *A* is the radiation area, *θ* is the local zenith angle, *T*_s_ is the temperature covered by the sample, *T*_amb_ is the ambient temperature and assumed to be 300 K, *ε*(*λ, θ*) is the emissivity of the material at the wavelength *λ*. Atmospheric emissivity *ε*_atm_(*λ, θ*) is related to atmospheric transmittance, wavelength, and zenith angle, which can be obtained according to *ε*_atm_(*λ, θ*) = 1 – *t*(*λ*)^1/cos^ *^*θ*^*, where *t*(*λ*) is the atmospheric transmittance at zenith angle *θ*. In Equations (5) and (6), *I*_BB_ is the intensity of the radiation wave when the real‐time temperature is *T* and the wavelength *λ* generated by the black body, which is calculated by

(9) $$\begin{array}{c} I_{\mathrm{BB}}\;\left(T,\lambda \right)=\frac{2hc^{2}}{\lambda^{5}}\;\frac{1}{e^{hc/\lambda K_{B}T}-1} \end{array}$$

In Equation (7), the solar radiation intensity is provided by *I*_AM1.5_(*λ*), and *θ* is taken as 0° because our test device is completely facing the sun. Typical nonradiative heat transfer coefficient *h_c_* is in the range of 2–12 W m^−2^ K^−1^.

## Conflict of Interest

The authors declare no conflict of interest.

## Supporting information

Supporting Information

## Data Availability

Research data are not shared.
