# Supplementary material for: Dual‐Mode Integrated Janus Films with Highly Efficient NaH2PO2‐Enhanced Infrared Radiative Cooling and Solar Heating for Year‐Round Thermal Management
Source: Adv Sci (Weinh). 2023 Jan 13;10(7):2206176. doi: 10.1002/advs.202206176 (PMC9982563; doi:10.1002/advs.202206176)
Supplement: Supplementary file 1 — Supporting Information [file ADVS-10-2206176-s001.pdf]

ADVANCED SCIENCE
Open Access

Supporting Information

for *Adv. Sci.*, DOI 10.1002/advs.202206176

Dual-Mode Integrated Janus Films with Highly Efficient NaH_2PO_2-Enhanced Infrared Radiative Cooling and Solar Heating for Year-Round Thermal Management

Peng Yang, Jiajun He, Yanshan Ju, Qingyuan Zhang, Yipeng Wu, Zhengcai Xia, Liang Chen* and Shaochun Tang**

Supporting Information

Dual-mode integrated Janus films with highly efficient NaH_2PO_2-enhanced infrared radiative cooling and solar heating for year-round thermal management

Peng Yang,[1,2,3] Jiajun He,[2] Yanshan Ju,[2] Qingyuan Zhang,[2] Yipeng Wu,[2] Zhengcai Xia,*[,1] Liang Chen,*[,1] and Shaochun Tang*[,2,3]

[1] *School of Physics and Wuhan National High Magnetic Field Center, Huazhong University of Science and Technology, Wuhan, 430074, P. R. China.*

[2] *National Laboratory of Solid State Microstructures, Collaborative Innovation Center of Advanced Microstructures, Jiangsu Key Laboratory of Artificial Functional Materials, College of Engineering and Applied Sciences, Nanjing University, Nanjing 210093, P. R. China.*

[3] *Haian Institute of High-Tech Research, Nanjing University, Jiangsu 226600, P. R. China.*

Corresponding authors:

Z.C. Xia, xia9020@hust.edu.cn; L. Chen, liangchen@hust.edu.cn; S.C. Tang, tangsc@nju.edu.cn.

Table.S1 Comparison of the average solar reflectance $\bar{R}_{solar}$, average infrared emittance $\bar{\varepsilon}_{IR}$ and sub-ambient cooling temperatures of cooling side with other previously reported porous films.

Samples	$\bar{R}_{solar}$	$\bar{\varepsilon}_{IR}$	Cooling temperature	Reference
PMMA/NaH_2PO_2 porous film	92.6%	97.2%	8.8 °C	This work
3DPCA/SiO_2 film	96%	95%	8.6 °C	[1]
PVDF/PDMS porous film	97%	96%	12.3 °C	[2]
EPDM/SiO_2 porous film	96%	95%	12 °C	[3]
Nanoporous $MgHPO_4 \cdot 1.2H_2O$ powder	92%	94%	4.1 °C	[4]
3DPCA/TiO_2 film	97%	96%	10 °C	[5]
Hierarchical pores P(VdF-HFP) film	96%	96%	7 °C	[6]
3D PCA/h-$AlPO_4$ nano-laminated film	97%	96.6%	13 °C	[7]
SiO_2@PDMS film	85%	95%	2 °C	[8]

Table.S2 Comparison of the average solar absorbance $\bar{\alpha}_{solar}$ and heating temperatures of heating side with other previously reported photothermal material.

Samples	$\bar{\alpha}_{solar}$	Temperature under 1 Sun	Heating temperature	Reference
PPy modified cotton	98.0%	88 °C	49.5、45.5	This work
CNT@PDMS film	95%	36 °C	7 °C	[8]
PPy@PVDF-HFP	96.9%	60 °C	35.8	[9]
CNT embedded PAN nonwoven fabrics	90.8%	43 °C	23.7 °C	[10]
Ag NPs encapsulated in amorphous carbon	92.3%	94 °C	/	[11]
$Ti_3C_2T_x$ MXene Decorated nanoPE Textile	91.3%	/	43.7 °C	[12]
3D structured graphene metamaterial	95.0%	52 °C	/	[13]

References

[1] B. Xiang, R. Zhang, Y. Luo, S. Zhang, L. Xu, H. Min, S. Tang, X. Meng, *Nano Energy* **2021**, *81*, 105600.

[2] M.C. Huang, C.H. Xue, J. Huang, B.Y. Liu, X.J. Guo, Z.X. Bai, R.X. Wei, H.D. Wang, M.M. Du, S.T. Jia, Z. Chen, Y. Lai, A hierarchically structured self-cleaning energy-free polymer film for daytime radiative cooling, Chem. Eng. J. **2022,** 442, 136239.

[3] H. D. Wang, C. H. Xue, X. J. Guo, B. Y. Liu, Z. Y. Ji, M. C. Huang, S. T. Jia, *Appl. Mater.*

Today **2021**, *24*, 101100.
[4] X. Huang, N. Li, J. Wang, D. Liu, J. Xu, Z. Zhang, M. Zhong, *ACS Appl. Mater. Interfaces* **2020**, *12*, 2252.
[5] X. Chen, M. He, S. Feng, Z. Xu, H. Peng, S. Shi, C. Liu, Y. Zhou, *Opt. Mater.* **2021**, *120*, 111431.
[6] C. Feng, P. Yang, H. Liu, M. Mao, Y. Liu, T. Xue, J. Fu, T. Cheng, X. Hu, H. J. Fan, K. Liu, *Nano Energy* **2021**, *85*, 105971.
[7] S. Feng, Y. Zhou, X. Chen, S. Shi, C. Liu, T. Zhang, *J. Mater. Chem. A* **2021**, *9*, 25178.
[8] B. Dai, X. Li, T. Xu, X. Zhang, *ACS Appl. Mater. Interfaces* **2022**, acsami.2c01370.
[9] B. Xiang, R. Zhang, X. Zeng, Y. Luo, Z. Luo, *Adv. Fiber Mater.* **2022**.
[10] B. Zhu, H. Kou, Z. Liu, Z. Wang, D. K. Macharia, M. Zhu, B. Wu, X. Liu, Z. Chen, *ACS Appl. Mater. Interfaces* **2019**, *11*, 35005.
[11] W. Li, C. Wang, J. Yang, J. Wang, W. Zhang, *J. Mater. Chem. A* **2021**, *9*, 11300.
[12] M. Shi, M. Shen, X. Guo, X. Jin, Y. Cao, Y. Yang, W. Wang, J. Wang, *ACS Nano* **2021**, *15*, 11396.
[13] K.-T. Lin, H. Lin, T. Yang, B. Jia, *Nat. Commun.* **2020**, *11*, 1389.

Table.S3 The thermal conductivities of cotton and cooling/heating side of Janus film.

Sample	Heating side	Cooling side	Cotton
**Thermal conductivity (W/ mK)**	0.234	0.089	0.112

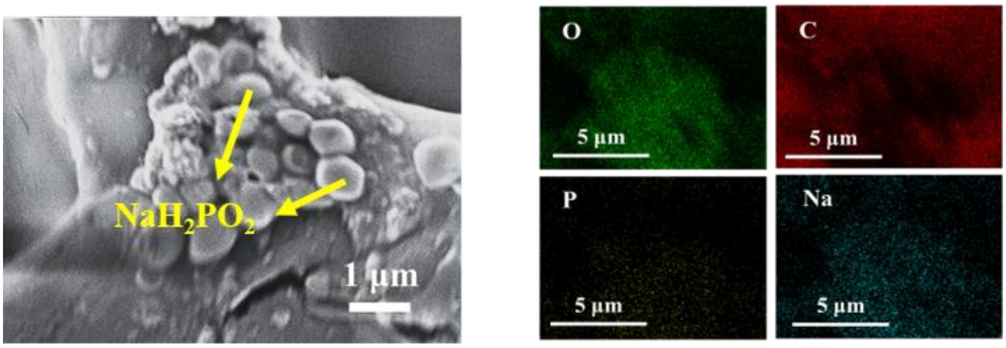

Figure S1. SEM images and EDS elemental mappings of individual NaH_2PO_2 particles**.**

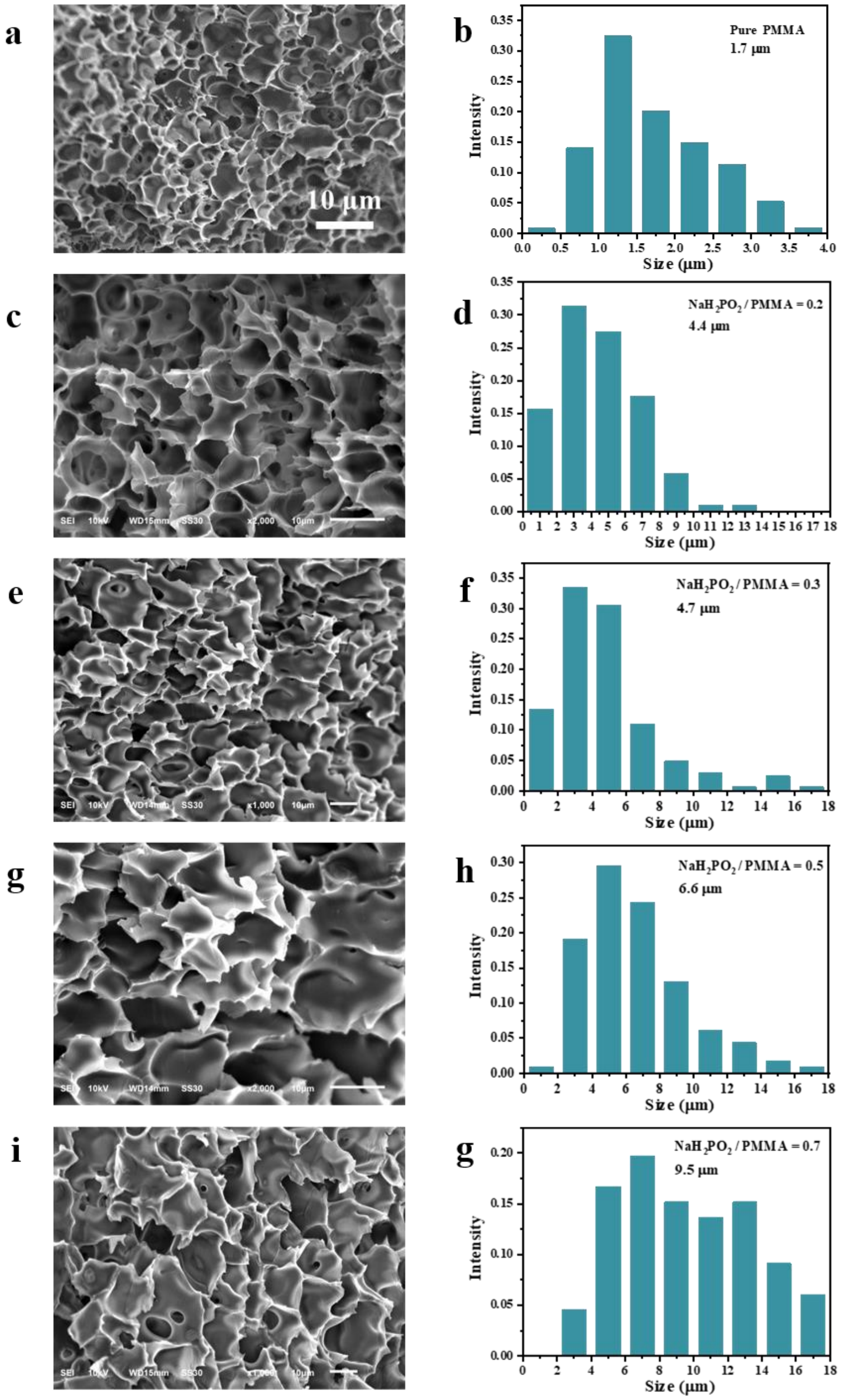

Figure S2. SEM images of cross section and micropore size distribution for (a-b) Pure PMMA, (c-d) PMMA/NaH_2PO_2-0.2, (e-f) PMMA/NaH_2PO_2-0.3, (g-h) PMMA/NaH_2PO_2-0.5 and (i-j) PMMA/NaH_2PO_2-0.7.

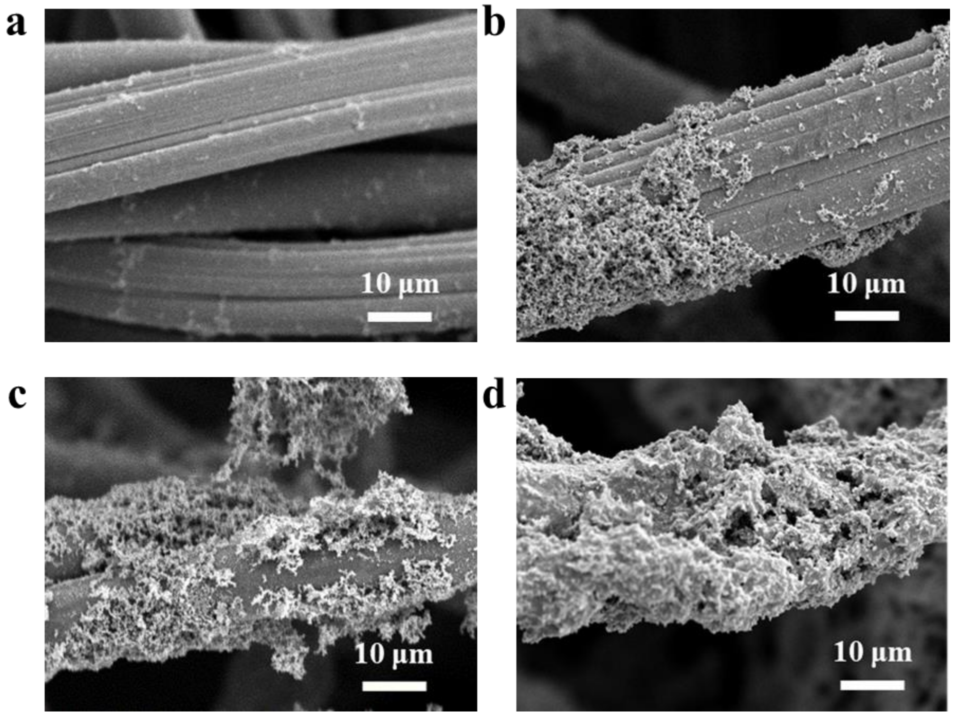

Figure S3. SEM images of PMC with different Fe^{3+} concentrations. (a) PMC-0.5 M, (b) PMC-1 M, (c) PMC-2 M, (d) PMC-4 M.

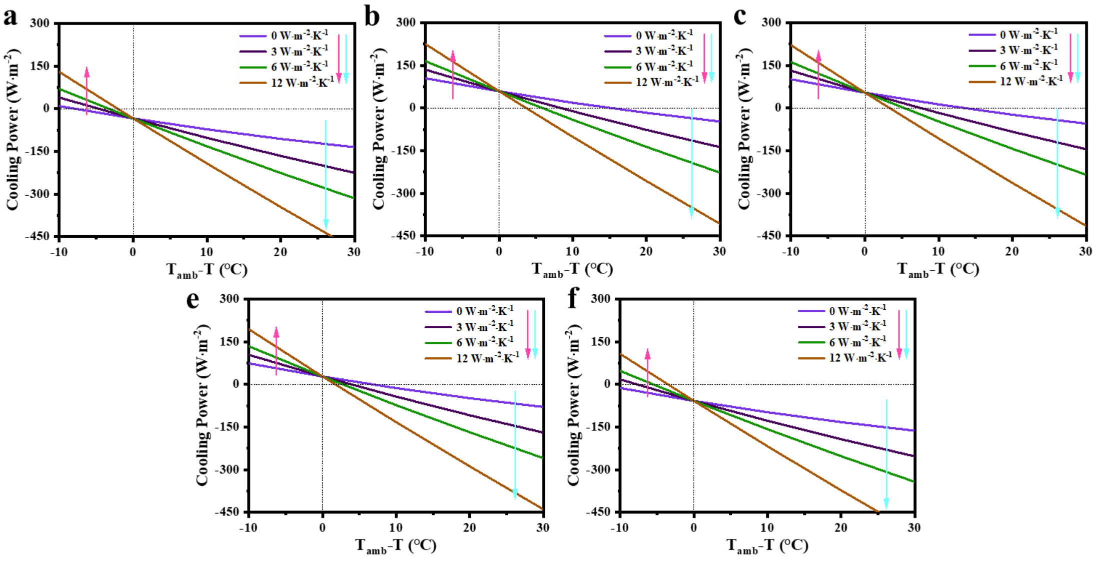

Figure S4. Calculated net cooling power of (a) PMMA, (b) PMMA/NaH_2PO_2-0.2, (c) PMMA/NaH_2PO_2-0.3, (d) PMMA/NaH_2PO_2-0.5 and (e) PMMA/NaH_2PO_2-0.7 during the daytime. The variable h_c is a combined nonradiative heat coefficient. Values of 0, 3, 6, 9, and 12 for h_c are used in the calculations.

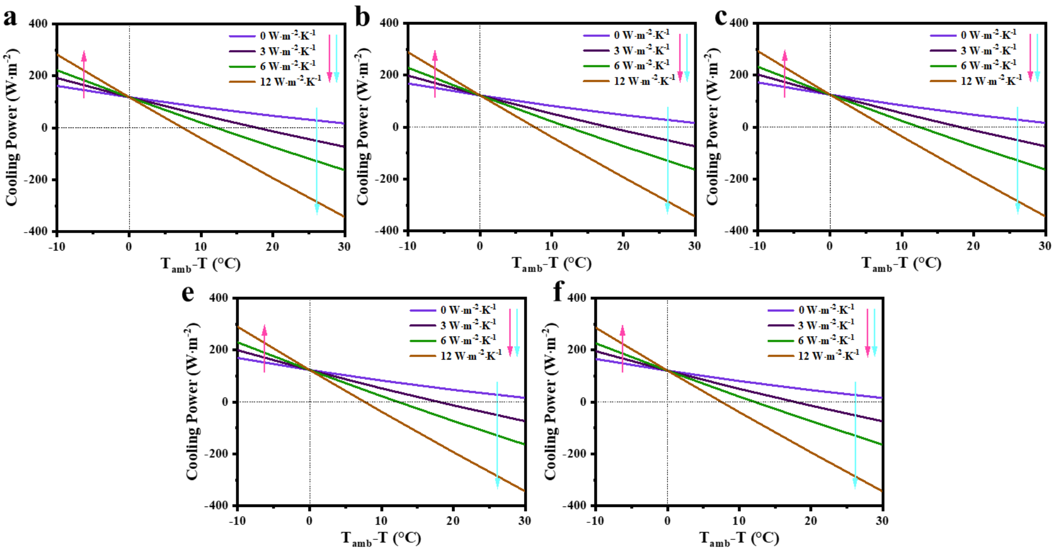

Figure S5. Calculated net cooling power of (a) PMMA, (b) PMMA/NaH_2PO_2-0.2, (c) PMMA/NaH_2PO_2-0.3, (d) PMMA/NaH_2PO_2-0.5 and (e) PMMA/NaH_2PO_2-0.7 during the nighttime. The variable h_c is a combined nonradiative heat coefficient. Values of 0, 3, 6, 9, and 12 for h_c are used in the calculations.

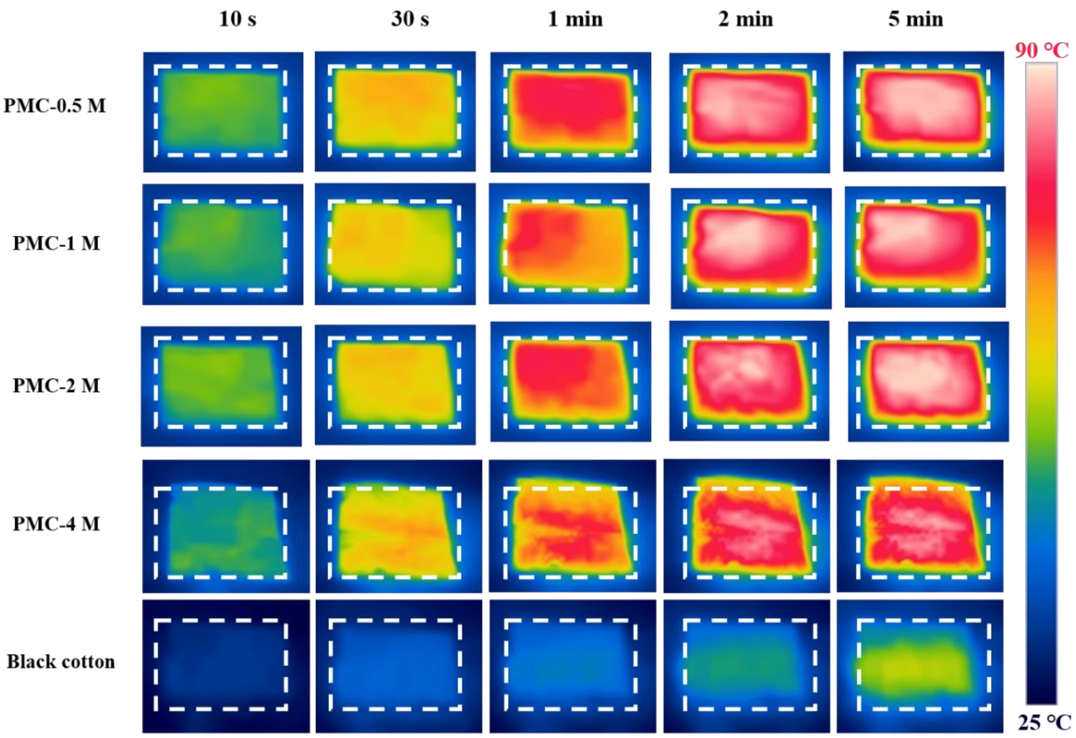

Figure S6. IR images for a series of PMCs and black cotton.

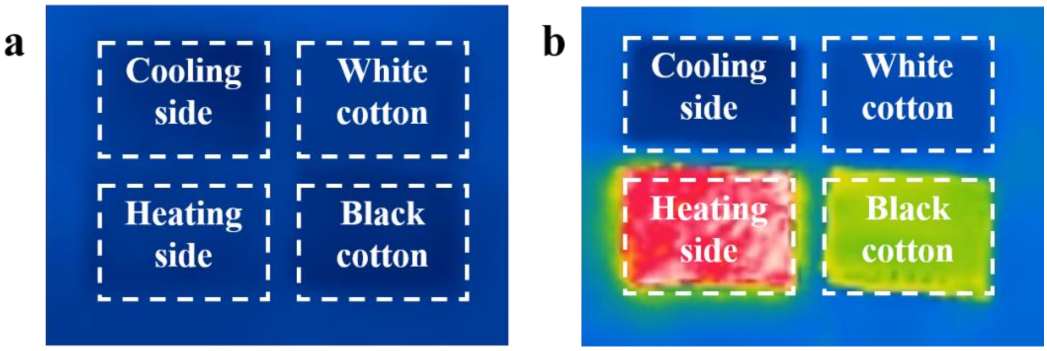

Figure S7. Infrared images of different samples under 100 mW·m^{-2} solar intensity.

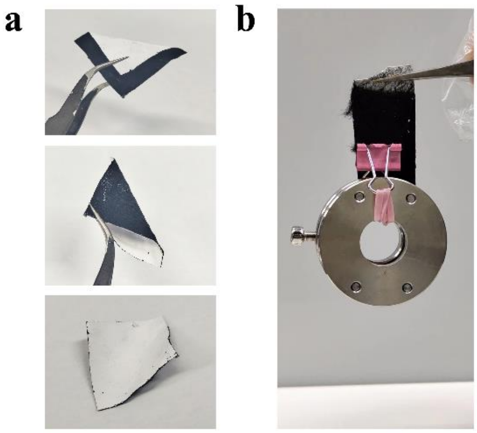

Figure S8. (a) Mechanical deformation and (b) adhesion of the dual-mode Janus film.

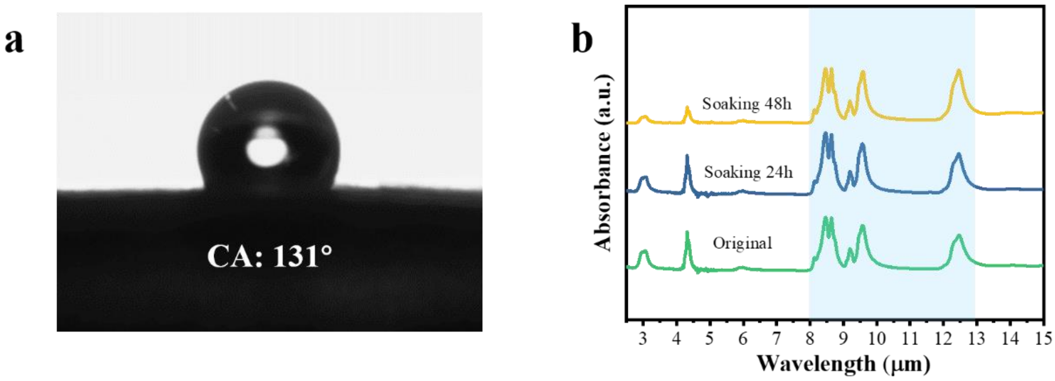

Figure S9. (a) Apparent water contact angles of cooling side. (b) Absorbance spectra of the cooling side after immersion in aqueous solutions.
